# Supplementary material for: Clinical Implementation of β-Tubulin Gene-Based Aspergillus Polymerase Chain Reaction for Enhanced Aspergillus Diagnosis in Patients with Hematologic Diseases: A Prospective Observational Study
Source: J Fungi (Basel). 2023 Dec 13;9(12):1192. doi: 10.3390/jof9121192 (PMC10744750; doi:10.3390/jof9121192)

Contents of Supporting information

Supplementary Methods……………………………………………………………………1

Table S1. Primer sequence used for the β-tubulin gene PCR assay…………………………4

Table S2. Results of *β-tubulin* gene-based *Aspergillus* PCR…………………………………5

Figure S1. PCR inhibitor effects on test results via SPUD assay……………………………7

Supplementary Methods

2.4 Fungal DNA extraction from bronchoalveolar lavage fluid

Clinical samples were isolated from the room temperature environment, aliquoted and stored at -80 °C until use in experiments. Before the experiment, the clinical sample was rapidly melted at 37 °C and used in the experiment. 1–3 mL of the BAL fluid sample was centrifuged at 10,000 × *g* for 5 min at room temperature, and the pellet and supernatant were separated and transferred to a new 1.5 mL tube. The BAL fluid sample used in the experiment is strictly ≥ 1mL, which is a condition considering the minimum detection limit included in the extracted DNA. The separated supernatant was stored at 4 °C to prevent degradation of suspended free DNA that may be included in the sample. Supernatant was included in the extraction process to extract DNA eluted from AFT or DNA containing an immune response exposed in a clinical state. This is a way to determine if the patient has been exposed to the fungus. After washing the pellet with 1 mL of PBS, 50 µL of PBS, and 5 µL of protease K were added, followed by a reaction at 65 °C for 30 min. Equal volumes of glass beads (1.0-mm zirconium beads; Sigma-Aldrich, St. Louis, MO, USA) were applied, followed by bead beating at maximum speed for five rounds of 1 min each. To prevent DNA denaturation, the tube was sufficiently cooled in a cold environment in the middle of bead beating. A total of 500 µL of the supernatant was added to the storage, pipetted more than 10 times, and transferred to a new tube. Subsequently, DNA extraction was performed using the High Pure PCR Template Preparation Kit (Roche, Basel, Switzerland), according to the manufacturer's protocol, and DNA was eluted in less than 100 µL for later use. DNA must be eluted in < 100

μL and is the minimum value to increase the number of DNA copies included per μL with the volume for the minimum detection limit. DNA extraction using bead beating is an excellent DNA extraction method for fungi and is a recommended approach in terms of time, stability, and efficiency Additionally, DNA can be extracted quickly and simply using other commercially available kits. As a method of eluting high-quality DNA, you can perform lysis using a DNA extraction buffer after bead beating and then use a phenol:chloroform:isoamyl alcohol solution. Although it satisfies high yield and quality, the complexity and harmfulness of the purification process cannot be ruled out due to the use of reagents such as phenol.

Afterwards, the following experiment was performed to confirm that DNA extraction was performed properly using the culture-negative BAL solution. After adding 10-fold diluted *A. fumigatus* conidia 1×10^5 to 1 mL of BAL solution and extracting *Aspergillus* DNA by the above method, probe real-time PCR was performed as follows. After sequentially diluting the *Aspergillus* positive control (APC) samples and performing PCR in the same manner, a standard curve was drawn to confirm that DNA was properly extracted from the conidia.

Table S1. Primer sequence used for the *β-tubulin* gene PCR assay

PCR	Target species	Primers / probes	Sequence (5′ → 3′)	Target loci	Product size
Multiplex real-time PCR primer	*Aspergillus spp.*	benA F3	TCGGTGTAGTGACCCTTGG	*benA*	254 ~ 272 bp
		benA R2	GCTGGAGCGYATGAACGTCT		
	A. fumigatus	TR F1	TAATCGCAGCACCACTTCAG	*cyp51A*	WT: 111 bp
		TR R1	AGGGTGTATGGTATGCTGGAA		TR34 : 145 bp
Hydrolysis probe	Ascomycetes	Asco 1F9	6-FAM-AVACGAAGTTGTCGGGRC- BHQ1	*benA*	18 bp
	Section *Fumigati*	Fumi 1R2	CY5-CGGCAACATCTCACGATCTGACTCGC-BHQ3		26 bp
	Section *Nigri*	Nig 1R26	HEX-ACTTCAGCAGGCTAGCGGTAACAAGT- BHQ1		26 bp
	Section *Flavi*	Flavi 1F18	HEX-CGGTCAGGAGTTGCAAAGCGTTTTCA-BHQ1		26 bp
	Section *Terrei*	Terrei 1R29	6-FAM-ACCATCCTGGGACAGATTCTYCACGC- BHQ1		26 bp
	A. tubingensis	Tub_1R21	CY5- AGGTTAGATCACACCGTCCCTGAGTT- BHQ3		25 bp

BenA, β-tubulin; bp, base pair; *cyp51A*, Cytochrome P450 Sterol 14-alpha demethylase; TR, tandem repeat; WT, wild type.

Table S2. Results of *β-tubulin* gene-based *Aspergillus* PCR

No.	Specimen No.	*Ascomycete*	*Fumigati*	*Nigri*	*Terrei*	*Flavi*	*A. tubingensis*
1	BF075						
2	BF076	+	+				
3	BF103						
4	BF110	+	+				
5	BF126						
6	BF134						
7	BF135						
8	BF136	+		+			
9	BF137	+				+	
10	BF191						
11	BF192						
12	BF209						
13	BF217						
14	BF218						
15	BF225						
16	BF232						
17	BF233						
18	BF234	+					
19	BF235						
20	BF240	+			+		
21	BF241	+				+	
22	BF245	+					
23	BF246	+					
24	BF247						
25	BF248						
26	BF251	+	+				
27	BF252						
28	BF256	+					
29	BF257						
30	BF258	+					
31	BF260	+				+	
32	BF261	+	+	+			+
33	BF262						
34	BF270	+		+			
35	BF275						
36	BF276	+		+			
37	BF277						
38	BF278						
39	BF279						
40	BF280	+	+				
41	BF286	+	+				
42	BF302	+				+	
43	BF303						
44	BF304						
45	BF305	+	+				
46	BF313						
47	BF314	+				+	
48	BF315						
49	BF323	+	+				
50	BF333	+		+			
51	BF334	+		+			
52	BF335	+	+				

53	BF348	+		+			
54	BF349	+		+			+
55	BF350	+		+			
56	BF360	+	+	+			
57	BF361	+		+			
58	BF362	+	+				
59	BF375	+					
60	BF376	+		+			
61	BF379						
62	BF383	+		+			
63	BF384	+	+	+			
64	BF392						
65	BF396	+		+			
66	BF397	+	+				
67	BF399						
68	BF402	+					
69	BF404						
70	BF405						
71	BF412						
72	BF417						
73	BF423						
74	BF424						
75	BF425						
76	BF437						
		Ascomycete	*Fumigati*	*Nigri*	*Terrei*	*Flavi*	*A. tubingensis*
	Result (Coinfection)	38	13 (3)	15 (3)	1	5	2

Figure S1. PCR inhibitor effects on test results via SPUD assay.

SPUD assay was performed to confirm the PCR inhibitor using 40 bronchoalveolar lavage (BAL) fluid and 20 DW samples. The results confirmed that the Cp value between the two PCRs differed within 1, which indicate that there was no PCR inhibitor in the BAL fluid sample or had no significant affect. Cp, crossing point.

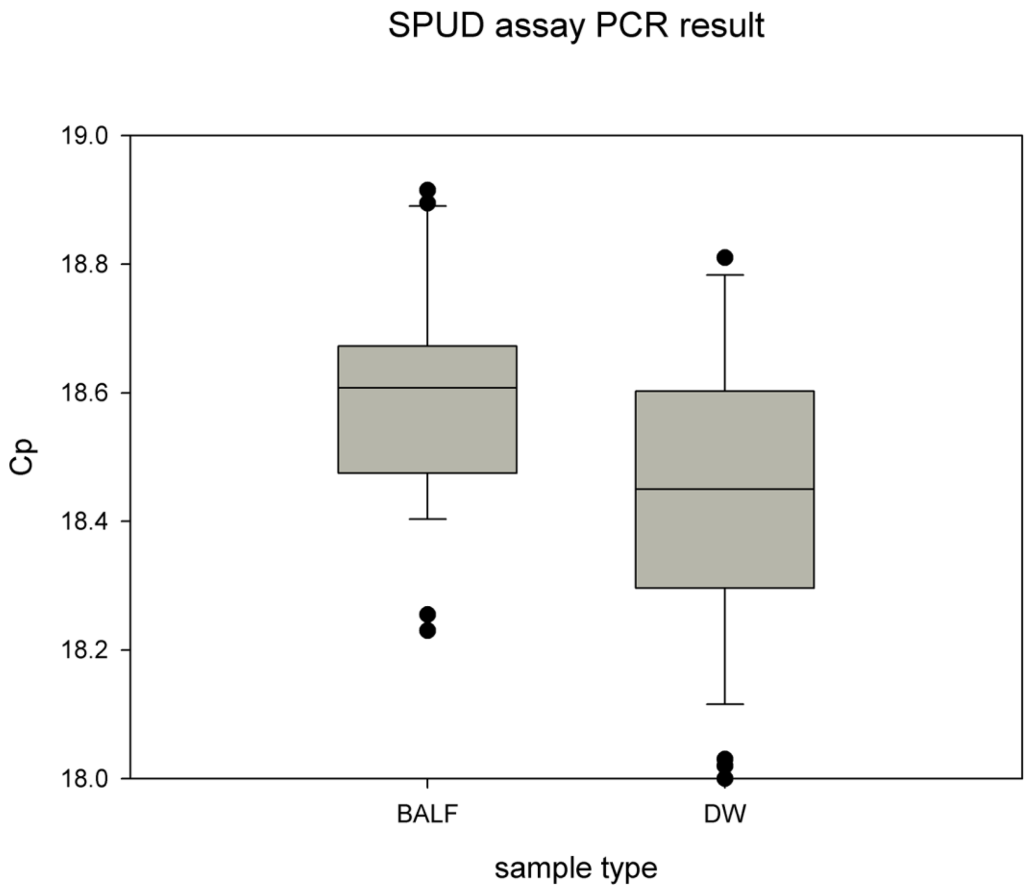

Supplement: Supplementary file 1 [file jof-09-01192-s001.zip › jof-2737911-supplementary.pdf]
